# Steroidogenic, Metabolic, and Immunological Markers in Dairy Cows Diagnosed With Cystic Ovarian Follicles at Early and Mid-Late Lactation

**DOI:** 10.3389/fvets.2019.00324

**Published:** 2019-09-26

**Authors:** Fabio S. Lima, Diego A. V. Acosta, Tonja R. Egan, Cassandra Skenandore, Saige Sulzberger, Dennis D. French, Felipe C. Cardoso

**Affiliations:** ^1^Department of Veterinary Clinical Medicine, University of Illinois, Urbana, IL, United States; ^2^Department of Comparative Biosciences, University of Illinois, Urbana, IL, United States; ^3^Department of Animal Sciences, University of Illinois, Urbana, IL, United States; ^4^Corporación Colombiana de Investigación Agropecuaria - Agrosavia, Bogota, Colombia

**Keywords:** ovarian cyst, cytokines, steroidogenic enzymes, metabolism, dairy cows

## Abstract

The etiology of cystic ovarian follicles (COF) remains a conundrum with steroidogenic, immunological, and metabolic dysfunctions linked to its development. Studies suggest that COF development may occur as a result of disruption of the insulin signaling pathway and the severity of a negative energy balance in dairy cows, but mid to late lactation cows diagnosed with COF are unlikely to have issues with energy metabolism. Herein, we characterized the mRNA expression of steroidogenic (*LHCGR, StAR, CYP11A1, 3*β*-HSD*, CYP19A), immunological (*IL-1*β, *IL-6, IL-8, TLR-4, TNF)*, and metabolic markers (*IGF-1, IRS1)* in follicular fluid; and plasma and follicular fluid levels of E2, IL-1β, glucose, and NEFA in early and mid-late lactation COF cows. Lactating dairy cows were diagnosed as having COF (*n* = 11, follicle >20 mm persistent for 7 days, absence of corpus luteum, and flaccid uterus) while 11 herdmates cycling with a dominant follicle were classified as the control cows. Cows diagnosed with COF were classified as early lactation (COF-E, *n* = 5) cows, <35 days in milk (DIM); or mid-late lactation (COF-M/L, *n* = 6), ≥118 DIM cows. Results revealed that mRNA expression *StAR* was greater (*P* < 0.01) in COF-E cows than COF-M/L cows and the control cows. The mRNA expression *CYP19A1* was lower (*P* < 0.01) in COF-E cows and COF-M/L cows than in the control cows. The mRNA expression *IL-6 and IRS-1* tended to be greater and lower, respectively, in COF-M/L cows compared to the control cows. The mRNA expression *IGF-1* was greater (*P* < 0.01) in COF-E and COF-M/L cows than in the control cows. The plasma and follicular fluid concentration of NEFA was greater (*P* < 0.05) in COF-E cows than in COF-M/L and the control cows. Cows with COF-E had disturbances in steroidogenic and metabolic markers, while cows with COF-M/L had steroidogenic, immunological, and metabolic dysregulations, suggesting that COF pathogenesis may vary between early and mid-late lactation dairy cows.

## Introduction

The etiology of cystic ovarian follicles (COF) remains a puzzle, but hormonal, immunological, and metabolic imbalances have been linked to its development (1–5). Cows diagnosed with COF have a dysfunctional hypothalamic-pituitary-ovarian axis signature that includes parabasal concentration of progesterone, increased peripheral estradiol levels, increased LH pulse frequency and amplitude, and reduced LH and FSH receptors that translate into a lack of LH surge and ovulation (6–10).

The steroidogenic markers associated with this dysfunctional signature is not well elucidated. Previous studies indicated a higher expression of 3β-HSD in estrogen-active cyst, but not young growing cysts (11). A more recent study found a higher or lower protein expression steroid acute regulatory protein (*StAR)*, cytochrome P450 aromatase (*CYP19A1)*, and 3β-hydroxysteroid dehydrogenase (*3*β*-HSD)* in the theca and granulosa cells of COF depending on the follicular stage of development (5). For example, the protein expression of *CYP19A1* was lower in the preantral follicles of COF cows than in control cows, but *CYP19A1* was higher in the antral follicles of COF cows than in the control cows suggesting that the criteria used for comparisons can impact the results and its interpretation for the development of COF (5). The number of days postpartum when COF are diagnosed, cyst turnover, and response to the therapy are revised and vary considerably (12). Thus, the characterization of steroidogenic markers between early and mid-late lactation cows developing COF might help to improve the understanding the differences in COF pathogenesis.

Another key dysregulation reported in cows with COF is the expression of immunological markers such as cytokines – key regulators of cell proliferation, follicular development, atresia, ovulation, steroidogenesis, and angiogenesis (13–15). Few investigations were performed with dairy cows, using either a model with induced follicle persistence or naturally occurring COF (3, 16, 17). The first study using a model of persistent follicles (5, 10 and 15 days of follicular persistence with day 0 as expected ovulation) revealed that interleukin 8 (*IL-8)* and tumor necrosis factor-α (*TNF)* was higher early (0 and/or 5 days of follicular persistence), and *IL-6* was higher from 0 to 10 days of persistence, in relation to dominant follicles from the control group (3). A second study of persistent follicles found that persistent follicles at different stages of persistence had a higher expression of IL-1 receptor antagonist (*IL-1RA*), *IL-1* receptor II (*IL-1RII*), and *IL-4* than the control group (17). The single study characterizing pro-inflammatory cytokines in cows with spontaneous COF, revealed that there was no differences in mRNA expression of *IL-1*β*, IL-6*, and *TNF* measured by real-time polymerase chain reaction between cystic follicles and dominant follicles, but the expression of these pro-inflammatory cytokines was higher in cystic follicles when measured by immunohistochemistry (16). Altogether, these studies suggest that more research is needed to elucidate the role of pro-inflammatory cytokines in the etiology of COF.

Follicular steroidogenesis and follicular cell proliferation are modulated by metabolic hormones such as insulin and insulin growth factor-1 *(IGF-1)* (18–20). A wealth of information has linked COF development with the disruption of the insulin signaling pathway, other critical metabolic sensors, and the severity of a negative energy balance in dairy cows (1, 4, 5, 21–23). It has been shown that cows developing COF have reduced insulin and non-esterified fatty acids (NEFA) and increased IGF-1 in the plasma during the weeks preceding cyst development (21–23). The follicular fluid concentration of IGF-1 have been reported to be reduced in induced and spontaneous COF (24) and the mRNA expression was decreased in induced but not in spontaneous COF (25). The expression of the insulin receptor and subunit alpha p85 of PI3K were reported to be lower in COF cows than in the tertiary follicles of the control cows (1). Another recent study detected that cystic cows' metabolic changes included lower plasma insulin, lower follicular fluid glucose and triacylglycerol, and higher follicular fluid NEFA, beta-hydroxybutyrate, and cholesterol (4). Early postpartum dairy cows undergo a negative energy balance and have higher odds of having metabolism disturbances. Despite all of the evidence of metabolic disorder in cows with COF, few studies have attempted to evaluate if these disturbances are concurrent and relate to steroidogenesis and immunological markers, and why cows in mid-late lactation develop COF despite not having the same metabolic challenges.

Cystic ovarian follicles remain a significant cause of reproductive failure in lactating dairy cows. This leads to economic losses due to an increase in the time required from calving to conception and culling rates, while the response to therapy is also highly inconsistent (12). Further elucidation of COF concurrent disturbances on steroidogenesis, pro-inflammatory cytokines, and energy metabolites in early and mid-late lactation dairy cows might allow the development of surrogate measures to mitigate the negative impact of COF and its unresponsiveness to therapy. There is, therefore, a critical need to characterize concurrent steroidogenesis, immunological, and metabolic markers of cows with spontaneous COF diagnosed at early and mid-lactation. We hypothesize that cows diagnosed with COF at early lactation have different steroidogenesis, pro-inflammatory, and metabolic disturbances than cows diagnosed with COF at mid-late lactation. The aims of the current study were to characterize the mRNA expression of steroidogenic, immunological, and metabolic markers in the follicular fluid of cows diagnosed with COF at early and mid-late lactation. We also assessed plasma and follicular concentrations of E2, IL-1β, glucose, and NEFA.

## Materials and Methods

### Ethics

All experimental procedures carried out in this study were approved by the Institutional Animal Care and Use Committees at the University of Illinois—Urbana-Champaign (Protocol # 14-283).

### Experimental Design and Reproductive Management

Lactating dairy cows from the University of Illinois' dairy herd were examined weekly by ultrasound, and a cohort of 11 cows classified as having COF (follicle >20 mm persistent for 7 days, absence of corpus luteum, and flaccid uterus) (26) were enrolled in the study. Cows diagnosed with COF were classified as early lactation cows (COF-E, *n* = 5)—diagnosed <35 days in milk (DIM) cows, or mid-late lactation cows (COF-M/L, *n* = 6)—detected ≥118 DIM. A group of 11 cows previously synchronized, that were confirmed to be cycling with a dominant follicle (presence of corpus luteum >16 mm and follicle >9 mm) were enrolled as the controls at 60 DIM. The reproductive management for the University of Illinois' dairy farm includes weekly examination by a University of Illinois Veterinarian starting at 21 DIM, using transrectal ultrasonography (Easy Scan, BCF Technology, Rochester, MN, USA) with a 7.5 MHz linear array probe.

Cows in the control group were synchronized with a modified Presynch-OvSynch as previously described (27). Briefly, cows received two injections of prostaglandin F2 alpha—PGF—(Lutalyse, 25 mg dinoprost tromethamine, Zoetis Animal Health, Kalamazoo, MI USA) at 34 ± 3 and 48 ± 3 DIM, then 12 days later at 60 ± 3 DIM began the OvSynch program. The program consists of a first GnRH at 60 ± 3 DIM, followed by PGF treatment 7 days later at 67 ± 3 DIM, a second GnRH 56 h after PGF at 69 ± 3 DIM, and then artificial insemination 16-−20 h later at 70 ± 3 DIM. Cows received an ultrasonography examination at the 60 ± 3 DIM, and only those with a corpus luteum larger than 16 mm and a follicle larger than 9 mm were eligible to be enrolled as the control for cows diagnosed as having COF. The goal was to assure that cows in the control group had an active corpus luteum and a dominant follicle.

### Cows Descriptive Characteristics

The average (range) follicle diameters retrieved at follicular aspiration were 42.2 mm (31.0–60.0 mm), 30.3 mm (25.0–40.0 mm), and 14.1 mm (9.0–19.0 mm), respectively, for the COF/E, COF-M/L and control cows. The average (range) days in milk were 32 days (30–34 days), 210 days (118–382 days), and 60 days (57–63 days), respectively, for the COF/E, COF-M/L, and control cows. The daily milk production averages (ranges) in the month of diagnosis were 41.6 kg (25.6–54.8 kg), 32.1 kg (7.6–52.6 kg), and 39.3 kg (22.1–50.7 kg), respectively, for the COF/E, COF-M/L, and control cows. The average number of lactations were 2.8 (1–6), 2.3 (1–4), and 2.7 (2–6), respectively, for the COF/E, COF-M/L, and control cows.

### Ovum Pick-Up for Follicular Fluid Collection

The follicle of COF cows and the dominant follicles of the control cows were aspirated to collect follicular fluid and cells. The control group had the dominant follicle aspirated at 60 ± 3 DIM. Briefly, the area above the first intercoccygeal space was clipped and disinfected using gauze embedded with an iodine solution and 70% ethanol. Then the first intercoccygeal space was injected with 5 ml of lidocaine (2% lidocaine hydrochloride solution, Hospira, Inc., Lake Forest, IL, USA) and 5 min were allowed to let the anesthesia to take effect. The vulva and perineal area were cleaned and disinfected with gauze embedded in an iodine solution. The ultrasound scanner equipped with a 7.5 MHz curvilinear probe (IBEX, E.I. Medical Imaging, Loveland, Colorado) was used for the follicle aspiration procedure. The ultrasound probe was placed within a custom-made handle. The handle enclosed the probe cord and fixed the head of the probe at a 30° angle relative to the needle guide. The device received a sterile lubricant and was introduced in the vagina slightly posterior to the cervix, toward the ovary containing the follicle that was re-measured and aspirated. The aspiration needle (18 G, WTA, Cravinhos, SP, Brazil) was guided through the stroma of the ovary and into the dominant follicle or cyst. The follicular fluid was collected and frozen at −80°C for a subsequent analysis of hormones. The follicular cells were immediately pelleted by centrifugation, at 4°C for 10 min at 2,000 × g and the follicular fluid was transferred. The cells were homogenized with Qiazol reagent (Qiagen, Hilden, Germany), snap-frozen in liquid nitrogen, and stored at −80°C.

### Markers Selection, RNA Extraction, and Quantitative PCR

The rationale for the markers investigated in the current study was to concurrently analyze genes that are the hallmark of an active innate immune response (*IL-1*β*, IL-6, IL-8, TLR-4, TNF*), genes that represent key steps of steroidogenic cascade (*LHCGR, StAR, CYP11A1, 3*β*-HSD, CYP19A*), and essential metabolic markers (IGF-1, IRS1) in two types of COF cows (early and mid-late lactation) that have a different metabolic profile. These genes have been reported to be altered in COF cows as described in the introduction, but an integrative empirical approach could shed light if discrepancies between key disrupted biological pathways of COF pathogenesis in early postpartum and mid to late lactation exist.

The RNA of follicular cells was extracted using the miRNeasy kit (Qiagen, Hilden, Germany), according to manufacturer's protocols, as detailed in our previous study (28). Quantitative PCR was performed using 4 ml diluted cDNA combined with 6 ml of a mixture composed of 5 ml of SYBR Green master mix (Quanta Biosciences, Gaithersburg, MD, USA), 0.4 ml each of 10 mm forward and reverse primers, and 0.2 ml DNase/RNase free water in a MicroAmpTM Optical 384–Well Reaction Plate (Applied Biosystems, CA) as detailed in our previous study (28).

Primers were designed using Primer Express 2.0 with a minimum amplicon size of 80 bp (when possible amplicons of 100–120 bp were chosen) and limited 30 G þ C (Applied Biosystems) (Table 1). When possible, primer sets were designed to fall across exon-exon junctions. Primers were aligned against publicly available databases using BLASTN at NCBI (29) and the UCSC's Cow (*Bos taurus*) Genome Browser Gateway. Before qPCR, primers were tested in a 20 μl PCR reaction using the same protocol described for qPCR excepting for the final dissociation protocol. For primer testing we used a universal reference cDNA (RNA mixture from different bovine samples) to ensure identification of desired genes. Five μl of the PCR product was run in a 2% agarose gel stained with SYBR safe. Only those primers that did not present primer-dimers and a single band at the expected size in the gel and those that had the right amplification product (verified by sequencing) were used for qPCR. The accuracy of a primer pair also was evaluated by the presence of a unique peak during the dissociation step at the end of qPCR. The extraction and qPCR analysis was performed using previously established protocols at Dr. Loor's Lab at the University of Illinois (30, 31). The final data were normalized using the geometric mean of GAPHD, β-ACT, and H2AFZ, which were validated as suitable internal control genes in bovine liver and adipose tissue.

**Table 1:** List of forward and reverse primers used in real-time PCR analysis.

**Gene**	**Primer**	**Primer sequence (5^′^–3^′^)**	**bp**	**Accession number**
*LHCGR*	Forward	AGAACACTAAAAACCTGGTGCACAT	100	NM_174381.1
	Reverse	GGAAGCTTGTGGATGCCTGTA		
*StAR*	Forward	TGGCATGGCCACACTCTATG	118	NM_174189.2
	Reverse	TGAGAAGTGCTGATGTACCA		
*CYP11A1*	Forward	CGTCAGCCTCCTGCACAAG	100	NM_174093.1
	Reverse	TTCTCGTCACTGTAGTAAGCCATCA		
*3β-HSD*	Forward	TGTCATTGACGTCAGGAATGC	100	NM_176644.2
	Reverse	TACGCTGGCCTGGACACA		
*CYP19A1*	Forward	AGCATAGATTTCGCCACTGAGTT	100	NM_174305.1
	Reverse	GCGCTGCGATCAGCATTT		
*IL-1β*	Forward	ATTCTCTCCAGCCAACCTTCATT	100	NM_174093.1
	Reverse	TTCTCGTCACTGTAGTAAGCCATCA		
*IL-6*	Forward	CCAGAGAAAACCGAAGCTCTCAT	100	NM_173923.2
	Reverse	CCTTGCTGCTTTCACACTCATC		
*IL-8*	Forward	GACAGCAGAGCTCACAAGCATCT	105	NM_173925.2
	Reverse	AAGCTGCCAAGAGAGCAACAG		
*TLR-4*	Forward	TGCGTACAGGTTGTTCCTAACATT	109	NM_174198.6
	Reverse	TAGTTAAAGCTCAGGTCCAGCATCT		
*TNF-α*	Forward	CCAGAGGGAAGAGCAGTCCC	114	NM_173966.3
	Reverse	TCGGCTACAACGTGGGCTAC		
*IGF-1*	Forward	CCAATTCATTTCCAGACTTTGCA	103	NM_001077828.1
	Reverse	CACCTGCTTCAAGAAATCACAAAA		
*IRS-1*	Forward	TGTTGACTGAACTGCACGTTCT	112	XM_003585773.3
	Reverse	CATGTGGCCAGCTAAGTCCTT		
*GADPH*	Forward	TTGTCTCCTGCGACTTCAACA	103	NM_001034034.2
	Reverse	TCGTACCAGGAAATGAGCTTGAC		
*β-ACT*	Forward	ACCAACTGGGACGACATGGA	149	NM_173979.3
	Reverse	GTCTCGAACATGATCTGGGTCAT		
*H2AFZ*	Forward	GAGGAGCTGAACAAGCTGTTG	105	NM_174809.2
	Reverse	TTGTGGTGGCTCTCAGTCTTC		

### Blood and Follicular Fluid Metabolite Analyses

Blood samples were collected at the day of follicle aspiration using evacuated tubes (Becton Dickinson, Franklin Lakes, NJ) containing K2 EDTA for plasma separation. Samples were placed immediately on ice and kept refrigerated until arrival to the laboratory. Blood tubes were centrifuged at 2,000 × g for 15 min at 5°C, and an aliquot of 2 ml of plasma was frozen at −80°C until analysis. Concentrations of E2 were measured using radioimmunoassay according to previous procedures which were slightly modified (32–34). The modifications were the substitution of a second antibody precipitation procedure in place of the charcoal extraction procedure. We used 100 μl 3-iodo-estradiol-17β (4.0 pg/tube, Carson, CA, USA). For plasma estradiol a volume of 200 μl of plasma diluted with 3 ml of benzene was used: toluene (2:1 vol/vol) solvent, whereas for follicular fluid the assay was diluted 1:1000. The estradiol assay was sensitive to 0.5 pg/ml and had an intra-assay CV of 9% and an inter-assay CV of 11%. Plasma and follicular fluid concentration pro-inflammatory cytokine IL-1β was measured using a bovine ELISA (Cat. No. ESS0027; Thermo Scientific, Rockford, IL). Concentrations of NEFA and glucose on plasma and follicular samples were analyzed using commercial kits at the Veterinary Diagnostics Laboratory, College of Veterinary Medicine, University of Illinois.

### Statistical Analyses

A power analysis was performed to identify the required number of cows for the study using G Power^*^Power version 3.1 for Mac (Universität Düsseldorf, Germany). We based the power calculation on a previous study that reported a ~6-fold change decrease in *CYP19A1* mRNA expression in COF cows when compared with cows with large antral follicles (5). Accordingly, we anticipate a 6-fold reduction of the *CYP19A1* mRNA expression in COF cows when compared to cyclic herdmates having large pre-antral follicles. Thus, using a one tailed-test, a 5% type I error, and a power of 80%, a sample size of five cows per group was deemed necessary. Models included the main effect of the group (COF-E, COF-M/L, and control group) and the variables of interest mRNA expression of steroidogenic (LHCGR, StAR, CYP11A1, 3β-HSD, CYP19A), immunological (IL-1β, IL-6, IL-8, TLR-4, TNF), and metabolic markers (IGF-1, IRS1) in follicular fluid; and plasma and follicular fluid levels of E2, IL-1β, glucose, and NEFA. All models were analyzed using the GLIMMIX procedure of SAS version 9.4 (SAS/STAT; SAS Institute Inc., Cary, NC). Data were analyzed using models that fit a Gaussian or a logarithmic distribution, and residuals were assessed for normality. The covariance structure that resulted in the smallest Bayesian information criterion was selected. Relative expression values were obtained by raising the PCR amplification values to the power of delta-delta threshold cycle (ΔΔCT) obtained from ΔCT least-square mean differences of pairwise comparisons among treatments and controls as the reference group (35). The ΔCT values, subjected to statistical analysis, were generated by normalization of CT values from target genes with the geometric mean of CT values from reference genes as previously described (36). Correlation analyses were performed for the plasmatic and follicular fluid concentration of E2, glucose, NEFA, and IL-1 β. Confidence intervals for graphical representation of relative expression were generated from the lower and upper confidence limits obtained for ΔCT least-square mean differences as described by Yuan et al. (35). Differences with *P* < 0.05 were considered significant and 0.05 < *P* ≤ 0.10 were considered to be a tendency toward a statistical difference.

## Results

### LHCGR, StAR, CYP11A1, 3β-HSD, CYP19A1 mRNA Expression

**Figure 1:**
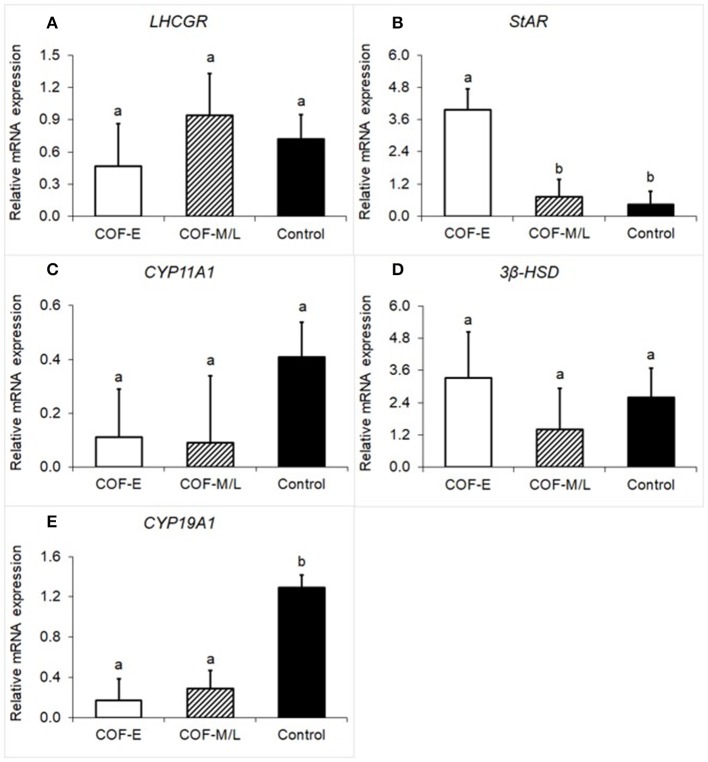
Fold-change in mRNA expression for five steroidogenic enzyme genes **(A)** *LHCGR*, **(B)** *StAR*, **(C)**, *CYP11A1*, **(D)** *3*β*-HSD*, and **(E)** *CYP19A1* detected in follicular fluid in cows diagnosed with COF at early (COF-E) and mid-late (COF-M/L) lactation and the control cows. Different letters (a,b) represent different (0.05 < *P* ≤ 0.10) least-square means. Error bars represent the mean standard error.

Cows diagnosed with COF-E and COF-M/L had a similar (*P* = 0.59) mRNA expression of luteinizing/choriogonadotropin hormone receptor (*LHCGR)* than that of the control herdmates (Figure 1A). Cow diagnosed with COF-E had a (*P* < 0.01) greater mRNA expression of *StAR* than that of COF-M/L and the control cows (Figure 1B). The mRNA expression of *CYP11A1* was similar (*P* = 0.35) in COF-E, COF-M/L, and the control herdmates (Figure 1C). Likewise, no differences (*P* = 0.69) in mRNA expression of *3*β*-HSD* in the COF cows and the control herdmates were detected (Figure 1D). The mRNA expression of *CYP19A1* was lower (*P* < 0.001) in the COF-E and COF-M/L cows than in the control herdmates (Figure 1E).

### IL-1β, IL-6, IL-8, TLR-4, TNF mRNA Expression

**Figure 2:**
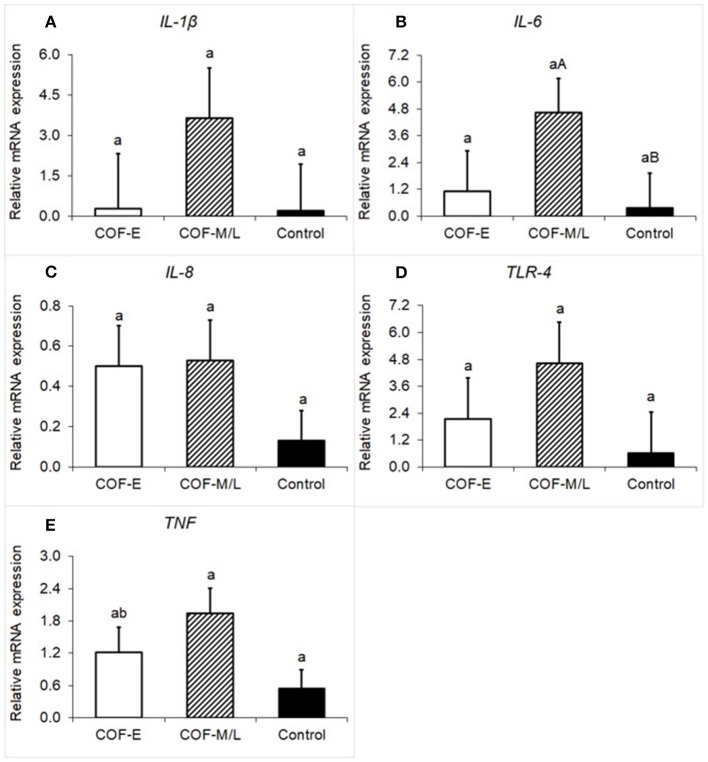
Fold-change in mRNA expression for five pro-inflammatory genes **(A)** *IL-1*β, **(B)** *IL-6*, **(C)** *IL-8* **(D)**, *TLR-4*, and **(E)** *TNF* detected in the follicular fluid in cows diagnosed with COF at early (COF-E) and mid-late (COF-M/L) lactation and the control cows. Different letters (a,b) represent different (*P* < 0.05) least-square means and capital letter **(A,B)** represent tendencies (0.05 < *P* ≤ 0.10). Error bars represent the mean standard error.

There were no differences (*P* = 0.36) for *IL-1*β mRNA expression between the COF-E, COF-M/L, and control cows (Figure 2A). Cows diagnosed with COF-M/L tend to have a higher mRNA expression (*P* < 0.09) of *IL-6* than the control cows, but no differences (*P* = 0.75) between COF-E cows and the control cows were detected (Figure 2B). There were no differences (*P* = 0.36) for *IL-8* mRNA expression between the COF-E, COF-M/L and control cows (Figure 2C). Likewise, no differences (*P* = 0.33) for *TLR-4* mRNA expression were detected between the COF-E, COF-M/L, and control cows (Figure 2D). The mRNA expression of *TNF* was greater (*P* = 0.03) in COF-M/L cows than in the control cows, whereas in the COF-E cows and control cows, TNF mRNA expression did not differ (*P* > 0.05) as depicted in Figure 2E.

### IGF-1 and IRS1 mRNA Expression

**Figure 3:**
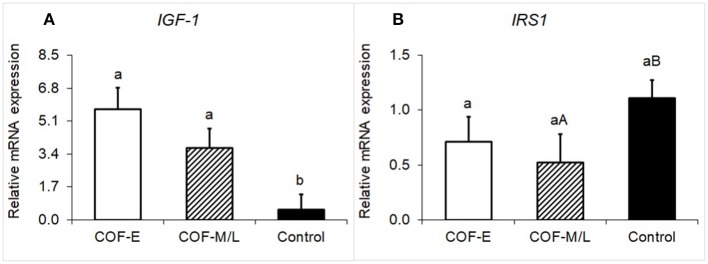
Fold-change in the mRNA expression for two energy metabolite genes **(A)** *IGF-1* and **(B)** *IRS1*, detected in the follicular fluid in cows diagnosed with COF at early (COF-E) and mid-late (COF-M/L) lactation and the control cows. Different letters (a,b) represent different (*P* < 0.05) least-square means and capital letter **(A,B)** represent tendencies (0.05 < *P* ≤ 0.10). Error bars represent the mean standard error.

Cows diagnosed with COF-E and COF-M/L had a greater (*P* < 0.02) mRNA expression of *IGF-1* than the control herdmates (Figure 3A). Moreover, cows diagnosed with COF-M/L tended to have a (*P* = 0.07) lower mRNA expression of *IRS1* than the control herdmates (Figure 3B), but COF-E cows and the control cows had similar (*P* = 0.17) mRNA expression (Figure 3B).

### E2, Glucose, NEFA, and IL-1β Concentrations in Plasma

**Figure 4:**
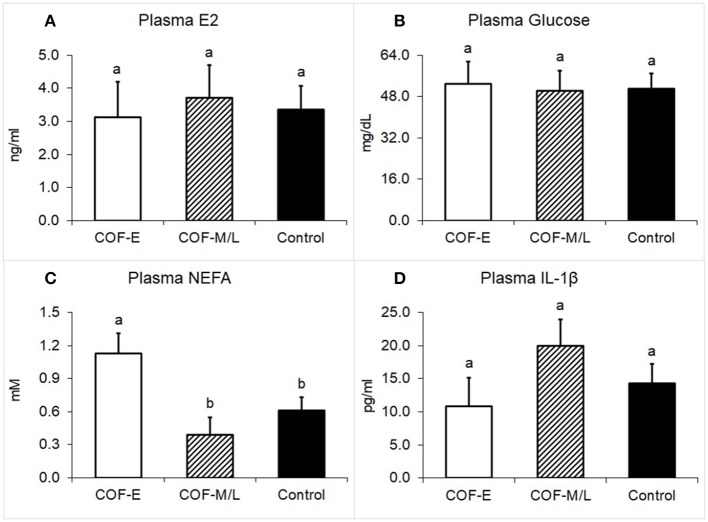
Plasma concentration for **(A)** E2, **(B)** Glucose, **(C)** NEFA, and **(D)** IL-1β in cows diagnosed with COF at early (COF-E) and mid-late (COF-M/L) lactation and the control cows. Different letters (a,b) represent different (*P* < 0.05) least-square means. Error bars represent the mean standard error.

Cows diagnosed with COF-E and COF-M/L had similar levels of E2 in the plasma (*P* = 0.92) than the control cows (Figure 4A). Likewise, the plasma concentration of glucose in cows diagnosed with COF in early and mid-late lactation did not differ (*P* = 0.97) from the control herdmates (Figure 4B). On the other hand, cows diagnosed with COF-E had a greater plasma concentration (*P* = 0.02) of NEFA than the COF-M/L and control cows (Figure 4C). The plasma concentrations of IL-1β did not differ (*P* > 0.05) between the COF-E, COF-M/L, and control cows (Figure 4D).

### E2, Glucose, NEFA, and IL-1β Concentrations in Follicular Fluid

**Figure 5:**
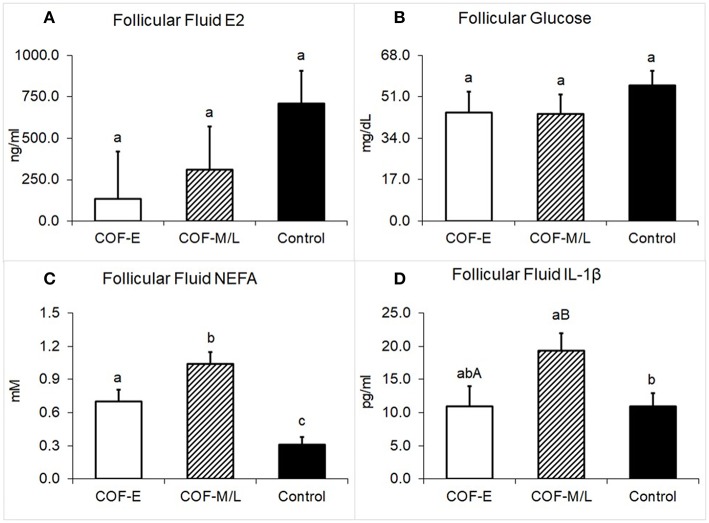
Follicular fluid concentration for **(A)** E2, **(B)** Glucose, **(C)** NEFA, and **(D)** IL-1β in cows diagnosed with COF at early (COF-E) and mid-late (COF-M/L) lactation and the control cows. Different letters (a,b) represent different (*P* < 0.05) least-square means and capital letter **(A,B)** represent tendencies (0.05 < *P* ≤ 0.10). Error bars represent the mean standard error.

Cows diagnosed with COF-E and COF-M/L had similar levels of E2 in the follicular fluid (*P* = 0.38) than the control cows (Figure 5A). The concentration of glucose in follicular fluid of cows diagnosed with COF-E and COF-M/L was similar (*P* = 0.40) to that of the control cows (Figure 5B). Cows diagnosed with COF-E had a higher concentration in the follicular fluid of NEFA (*P* < 0.001) than the control cows and a lower follicular fluid concentration of NEFA than the COF-M/L cows (Figure 5C). Also, the NEFA concentration in the follicular fluid of the COF-M/L cows was greater (*P* = 0.02) than in the control herdmates (Figure 5C) The follicular fluid concentration of IL-1β tended to be greater for the COF-M/L cows (*P* = 0.05) than for the COF-E cows, and the follicular fluid concentration of IL-1β was greater (*P* = 0.02) in the COF-M/L cows than in the control cows (Figure 5D). However, no differences (*P* = 0.98) in the follicular fluid concentration of IL-1β between the COF-E and control cows were detected (Figure 5D).

### Correlation of Plasma and Follicular Fluid Concentration of E2, Glucose, NEFA, and IL-1β

An analysis of associations for plasma and follicular fluid for metabolites E2, glucose, NEFA, and IL-1β in the COF-E, COF-M/L, and control cows revealed no significant relationship between follicular and plasma concentrations of these metabolites (Table 2).

**Table 2:** Correlation analysis of the plasma and follicular fluid concentration of E2, glucose, NEFA, and IL-1β in cows diagnosed with COF at early (COF-E) and mid-late (COF-M/L) lactation and the control cows.

**Groups**	**Metabolites measured in plasma and follicular fluid**							
	**E2**		**Glucose**		**NEFA**		**IL-1β**	
	**Corr**.	***P*-value**	**Corr**.	***P*-value**	**Corr**.	***P*-value**	**Corr**.	***P*-value**
COF-E	0.16	0.98	0.61	0.28	0.57	0.31	0.03	0.96
COF-M/L	−0.15	0.77	0.12	0.81	0.33	0.52	0.16	0.76
Control	−0.36	0.27	0.16	0.64	0.31	0.35	0.42	0.19

*Corr., Correlation between follicular fluid and plasma concentrations*.

## Discussion

As hypothesized, concurrent dysregulation of steroidogenic, immunological, and metabolic markers occurred in cows diagnosed with spontaneous COF and the time in lactation for diagnosis influenced the gene expression, plasma, and follicular fluid concentration disturbances found in the current study. Amongst the steroidogenic markers, differences between COF cows and the control cows were found only for *StAR* and *CYP19A1*. Interestingly, the mRNA expression of *StAR* was increased in COF cows at early but not mid-late lactation when compared to the control cows. Similarly, a recent study examined the mRNA expression of *StAR* in theca and granulosa and theca cells in small (<5 mm), medium (5–10 mm) and large antral follicles (>10 mm) with COF. It was reported that the *StAR* protein expression was higher in the granulosa cells of small antral follicles of cows with COF than in those of control cows (5). Another study that classified COF in cysts and young cysts (follicular structures >15 mm within 3–5 day of removal of previous cyst) reported an increase in *StAR* mRNA expression in the granulosa cells of cows with cysts, but not with young cysts, suggesting that the stages of development of the cyst might influence the characterization of steroidogenic disturbances reported in COF (37). Altogether, these previous findings along with ours, suggest that *StAR*, a limiting step in the biosynthesis of steroid hormones that facilitates the translocation of cholesterol from the outer to the inner mitochondrial membrane (38), is either not compromised or overexpressed in cows diagnosed with COF. The discrepancies in the pathogenesis of COF might be related to the time postpartum in which the disease occurred.

Our findings for *CYP19A1* concurred with previous studies that evaluated the mRNA expression in the granulosa of large follicles in comparison to follicular cysts, confirming that cows diagnosed with COF, independent of the number of days postpartum for diagnosis, have a lower mRNA expression of *CYP19A1* (37, 39). It has been suggested that reduced mRNA expression of *CYP19A1* combined with the increased expression of *StAR*, a sign of potentially increased cholesterol availability, might indicate that cows with COF are undergoing luteinization (40–45). It is reasonable to speculate that the profile of steroidogenic disturbances in COF early postpartum, but not in mid-late lactation, is suggestive of increased luteinization and parabasal levels of progesterone found in some cases of cystic cows. However, progesterone was not measured in the current study and, therefore, we need to be careful to interpret these results. Indeed, the lack of progesterone analysis limited the ability of the current study to distinguish if cysts were estradiol-dominant, progesterone-dominant, or low-steroidogenic active cysts as previously reported (46).

Immunological marker disturbances were only present in mid-late lactation cows diagnosed with COF. Indeed, cows diagnosed with COF-M/L had increased mRNA levels of *TNF* and follicular fluid of IL-1β, and tendencies for increased mRNA expression of *IL-6* when compared to the control cows. Previous studies had shown that cytokines are master modulators of the follicle milieu and fate, controlling cell proliferation, follicular development, atresia, ovulation, steroidogenesis, and angiogenesis (13–15). Our findings are consistent with previous studies using persistent follicle models that reveal increased mRNA expression of IL-1RA, IL-1RII, IL-4, IL-6, IL-8, and TNF depending on the length of persistence (3, 17). A single study characterized pro-inflammatory cytokines in cows with spontaneous COF, revealing no differences in mRNA expression of IL-1β, IL-6, and TNF (16). In this study the number of days postpartum reported for the diagnosis of COF was 55 ± 9.3, and the results were consistent with the current findings for COF early postpartum (16). The similarities between the abovementioned study and our COF-E cows, and the discrepancy between COF-E and COF-M/L cows in the current study, may indicate that the number of days postpartum might be a factor to consider to fully characterize COF pathogenesis.

It is unclear which factors might trigger the increased pro-inflammatory makers of cows diagnosed with COF-M/L, but not in COF-E cows when compared to the control cows. A recent study revealed that cows diagnosed with COF had an increased presence of lipopolysaccharide (LPS) and an increased expression of TLR-4, with the latter being consistent with COF-M/L cows in the current study (39). Although we did not measure LPS in the present study, we speculate that COF-M/L cows might have had increased levels of LPS in the follicle milieu, causing the disturbing profile of inflammatory markers reported herein. A potential process for an increased accumulation of LPS in these mid-late lactation COF cows might include a leaky gut or bacterial infection at the mammary gland (47–49). Further research needs to investigate if factors such as mastitis and gastrointestinal integrity issues are associated with endotoxemia and the development of COF in mid-late lactation dairy cows.

Our findings for *IGF-1* in COF-E and COF-M/L cows differed from a previous study that reported no differences in *IGF-1* mRNA expression in cows with spontaneous COF and reduced mRNA expression of *IGF-1* when compared to induced COF (50). Ortega et al. (24) measured the follicular fluid concentration of IGF-1 and reported a decrease in IGF-1. On the other hand, increased plasma levels of IGF-1 have been reported to be consistently higher during the first 16 weeks postpartum (22). The relationship between plasma and follicular fluid concentration of IGF-1 is controversial. A study reported that the plasma and follicular fluid of IGF-1 are highly correlated (51), whereas other studies either indicated that IGF-I in follicular fluid was lower (52), or greater (24) than plasma IGF-I concentration. Unfortunately, we did not measure IGF-1 in the plasma to help elucidate the specific relationship in the current study. Altogether, our findings as well as those of previous studies, indicate that there is a dysregulation on *IGF-1*. However, the exact behavior of this metabolic sensor in cystic cows needs to be further investigated.

The decreased tendency for mRNA expression of *IRS-1* in COF-M/L cows is another finding in the current study that resembles a previous study (1). The last study indicated this insulin receptor (IRS-1) was reduced in cows with spontaneous COF when compared to other follicular structures (1). Insulin is a pivotal player involved with steroidogenesis, follicular development, and ovulation (53, 54). Indeed, insulin exerts its metabolic effect through the phosphatidylinositol 3-kinase (PI3K) pathway (55, 56). The regulatory PI3K subunit binds to phosphotyrosine residues on IRS-1 that operate like a docking system for molecules binding to and activating cellular kinases involved in downstream signaling pathways involved in follicular development (53, 55, 56). The downregulation of IRS-1 found in the earlier study (1) and the tendency for the decreased expression of current research might suggest that this receptor could be a factor contributing to a disturbance that leads to COF.

As expected, early postpartum but not mid-late lactation COF cows had increased plasmatic levels of NEFA when compared to control cows. Intriguingly, the follicular fluid concentrations of NEFA were increased in the COF-E and COF-M/L cows when compared to control cows. Early postpartum dairy cows are likely to be under a negative energy balance and have a metabolic profile with a disrupted insulin pathway (Low insulin and IGF-1) and increased lipolysis that result in increased plasmatic levels of NEFA (57, 58). Our findings, however, are consistent with a recent study that revealed increased follicular fluid levels of NEFA in cows diagnosed with COF, but no differences in the plasma concentration of NEFA (4). The abovementioned cows with COF were in lactation from between 173 and 255 days, which is consistent with the range for COF-M/L cows (118–382 DIM) in the current study and help to explain the present findings.

There has been a wealth of information in the literature showing the detrimental impact of NEFA for granulosa cell survival and proliferation, steroidogenesis, and follicular and oocyte development (59–62). It is, therefore, reasonable to speculate that the increased follicular concentration of NEFA found in COF cows in the current study may compromise the granulosa cell function and steroidogenesis. Our COF-E cows' levels of NEFA in the follicular fluid and plasma were similar to those reported previously (4). On the other hand, COF-M/L cows had greater levels of NEFA in the follicular fluid than in the plasma. It has been suggested that because VLDL-triacylglycerol is not capable of passing the follicle wall (63, 64), an increased follicular fluid concentration of NEFA might be a result of triacylglycerol hydrolysis, and its availability would favor its oxidation to obtain energy as shown in COF-M/L cows. In the current study, we found that COF-M/L cows had an increased expression of immunological markers, which might suggest an increased energy requirement in follicle milieu (65). The pattern of activated pro-inflammatory cytokines might be the driving factor for metabolic changes found in COF-M/L cows in the current study.

## Conclusions

The results of the present study confirm that concurrent disruptions in steroidogenic, immunological, and metabolic markers occur in cows diagnosed with COF and the pattern of dysregulation varies according to the number of days postpartum. Disturbances in cows diagnosed with COF at early postpartum were limited to steroidogenesis and metabolic markers, while COF-M/L cows had steroidogenic, immunological and metabolic marker disorders. Altogether, the current study provides evidence that the characterization of mechanisms associated with COF development in early and mid-late lactation needs to be further investigated to develop therapies that can mitigate the negative impact of COF in dairy cows with a distinct pathogenesis.

## Data Availability Statement

The datasets generated for this study are available on request to the corresponding author.

## Ethics Statement

The animal study was reviewed and approved by Institutional Animal Care and Use Committees at the University of Illinois—Urbana-Champaign.

## Author Contributions

FL analyzed the data and wrote the manuscript. FL, DA, and FC designed the study. FL and FC revised the manuscript. FL, DA, TE, CS, SS, DF, and FC participated in the acquisition of the data. DA, TE, CS, and SS performed the laboratory analysis.

### Conflict of Interest

The authors declare that the research was conducted in the absence of any commercial or financial relationships that could be construed as a potential conflict of interest.
